# The crystal structure of (3*Z*)-4-[(3-hy­droxy­phen­yl)amino]­pent-3-en-2-one: helices and π-stacking inter­actions of hy­dro­gen-bridged rings

**DOI:** 10.1107/S2056989026004068

**Published:** 2026-05-07

**Authors:** Jebreil Syed, Alen Hadzovic

**Affiliations:** a Department of Physical and Environmental Sciences, University of Toronto Scarborough, 1065 Military Trail, Toronto, Ontario, M1C 1A4, Canada; Universidade de Sâo Paulo, Brazil

**Keywords:** crystal structure, keto–amine, Hirshfeld surface analysis, hy­dro­gen bond, π–π stacking

## Abstract

The title com­pound, C_11_H_13_NO_2_, crystallizes in the monoclinic space group *P*2_1_/*c* with four mol­ecular units per unit cell and *Z*′ = 1. A strong intra­molecular N—H⋯O inter­action results in a six-membered hy­dro­gen-bridged ring. An inter­molecular O—H⋯O hy­dro­gen bond leads to a helix formation. The Hirshfeld surface analysis of inter­molecular inter­actions is pre­sent­ed.

## Abstract 2

The title com­pound displays a strong intra­molecular N—H⋯O inter­action resulting in a six-membered hy­dro­gen-bridged ring. An inter­molecular O—H⋯O hy­dro­gen bond leads to a helix formation. The Hirshfeld surface analysis of inter­molecular inter­actions is pre­sent­ed.

## 1. Chemical context

A direct condensation of acetyl­acetone (acac) with primary amines or di­amines provides a simple route to a broad family of potential coordination ligands (Hernández-Molina & Mederos, 2003 ▸). Particularly prominent are the nacnac ligands, in which both carbonyl groups of the keto tautomer are replaced by imine functionalities, for example, in reactions with aniline and its 2,6-dialkyl derivatives (Hadzovic & Song, 2008*a* ▸; Hadzovic & Song, 2008*b* ▸). Also common are the so-called ‘half units,’ *O*,*N*-donor ligands that retain one unreacted carbonyl group (Hernández-Molina & Mederos, 2003 ▸). Designing polydentate systems is straightforward for both nacnacs and half units when the amine com­ponent bears additional donor groups.

Despite their structural richness and widespread use in coordination chemistry, the free nacnac and half-unit ligands themselves have received com­paratively little crystallographic attention. This contrasts with classical Schiff bases, which require an aryl-substituted carbonyl group and have been extensively analysed for intra- and inter­molecular inter­actions (Dominiak *et al.*, 2003 ▸; Karabıyık *et al.*, 2012 ▸). To extend similar structural studies to half units and nacnacs, we report here the structure and detailed analysis of the condensation product of acac with one equivalent of 3-amino­phenol.

## 2. Structural commentary

**Figure 1:**
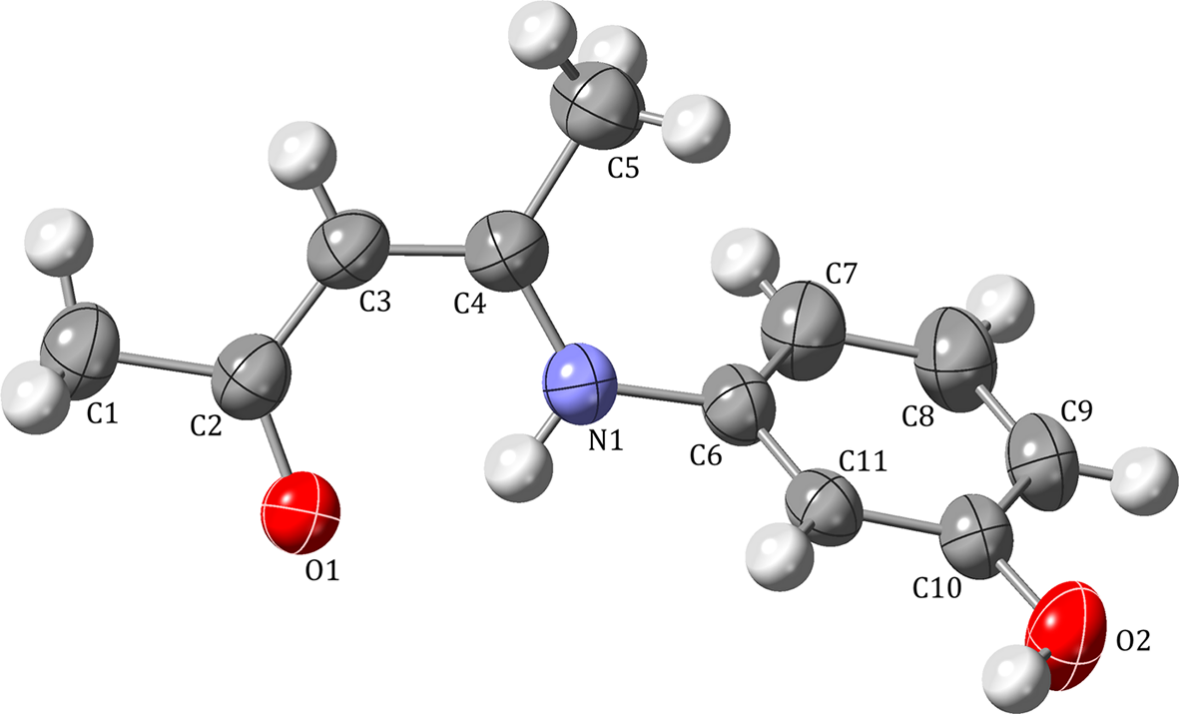
The mol­ecular structure and atom-labeling scheme for **I**, with displacement ellipsoids drawn at the 50% probability level.

The asymmetric unit of (3*Z*)-4-[(3-hy­droxy­phen­yl)amino]­pent-3-en-2-one (**I**) contains one independent mol­ecule (Fig. 1 ▸). The com­pound, formed through condensation of acetyl­acetone (acac) and 3-amino­phenol, exists as a keto–amine tautomer. Selected bond lengths and angles are provided in Table 1 ▸. Atoms O1, C2, C3, C4 and N1 are essentially coplanar (least-squares plane: −5.1098*x* + 7.0367*y* + 1.9389*z* = 2.6891, r.m.s. deviation = 0.0195 Å, the furthest atom from the plane is C2 at −0.0315 Å). Methyl atoms C1 and C5 lie −0.169 (2) and 0.0079 (17) Å, respectively, from this plane, while *ipso* atom C6 of the benzene ring is −0.1980 (16) Å away. The benzene ring is tilted by 20.38 (8)° with respect to the remainder of the mol­ecule.

A six-membered hy­dro­gen-bridged (HB) ring, a quasi-chelate ring that strongly resembles and can act as a covalently formed classical ring (Blagojević *et al.*, 2015 ▸), is formed through a strong intra­molecular N1—H1N⋯O1 hy­dro­gen bond (Table 2 ▸; for discussion of the remaining hy­dro­gen bonds, see *Supra­molecular features*) with *S*(6) graph-set notation (Etter, 1990 ▸). The bond lengths within the six-membered ring (Table 1 ▸) lie between typical single and double bonds for all bonding partners (Allen *et al.*, 1987 ▸). Together with the bond angles, these values are consistent with a heteroconjugated system and with N1—H1N⋯O1 as a resonance-assisted hy­dro­gen bond (RAHB; Bertolasi *et al.*, 1995 ▸; Filarowski *et al.*, 2005 ▸; Steiner, 2002 ▸). Elongation of the N1—C6 bond relative to typical N(amine)—C(aromatic) distances (1.355 and 1.394 Å for planar and pyramidal N atoms, respectively; Allen *et al.*, 1987 ▸) is also consistent with conjugation of the N-atom lone electron pair within the acac-derived backbone.

The aromaticity of this conjugated system was further assessed using Krygowski’s harmonic oscillator measure of aromaticity (HOMA) index (Krygowski, 1993 ▸): 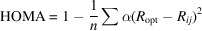
where *n* is the number of bonds, *R_ij_* and *R*_opt_ are experimental and optimal bond lengths, respectively, and α is a dimensionless normalization constant set to give HOMA value of 0 for a non-aromatic system and 1 for a fully aromatic one. Using the bond lengths from Table 1 ▸ and literature values for α (CC 257.7, CN 93.52 and CO 157.38; Krygowski, 1993 ▸; Krygowski & Cyrański, 2001 ▸) and *R*_opt_ (CC 1.388 Å, CN 1.334 Å and CO 1.265 Å; Krygowski, 1993 ▸; Krygowski & Cyrański, 2001 ▸) gives HOMA = 0.983, indicating a high level of aromaticity in **I**. In com­parison, the HOMA index for acetyl­acetone is 0.964 based on the single-crystal X-ray analysis data at 210 K from Boese *et al.* (1998 ▸).
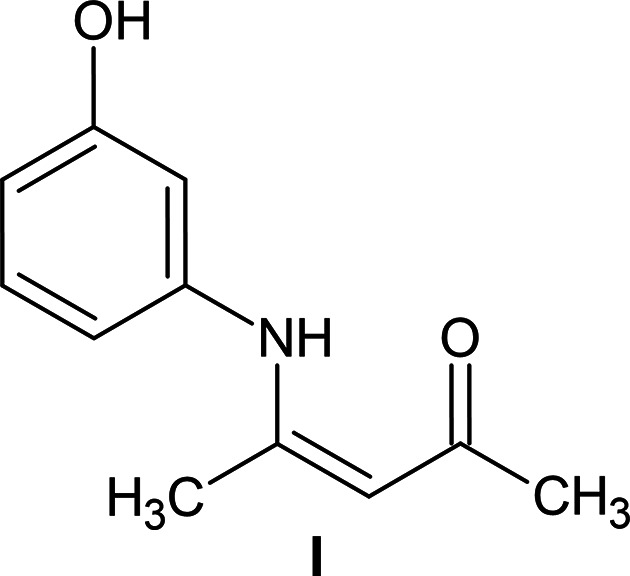


## 3. Supra­molecular features

**Figure 2:**
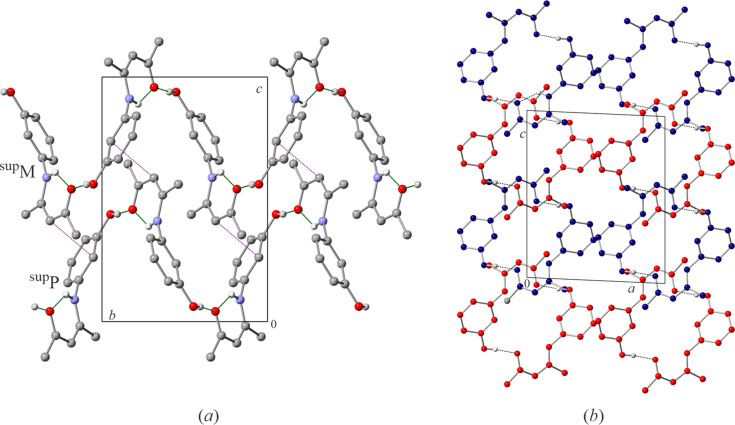
Compound **I** forms helices through intra­molecular hy­dro­gen bonds, (*a*) viewed along the crystallographic *a* axis, showing both left- and right-handed helices (^sup^M and ^sup^P, respectively) and (*b*) viewed along the crystallographic *b* axis emphasizing the packing of helices (blue are left- while red are right-handed). Green dashed lines indicate hy­dro­gen bonds (both intra- and inter­molecular), while purple dotted lines show short contacts between the helices.

The mol­ecules of **I** link *via* O2—H1O⋯O1^i^ [symmetry code: (i) −*x*, *y* + 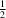, −*z* + 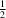] inter­molecular hy­dro­gen bonds to form helices parallel to the crystallographic *b* axis [Fig. 2 ▸(*a*)], corresponding to a *C*(10) graph-set assignment (Etter, 1990 ▸). The helices wrap around 2_1_ screw axes and have two mol­ecules per turn as imposed by symmetry. The left- and right-handed helices, ^sup^M and ^sup^P, respectively, inter­lock in each others grooves, forming a helix layer in the *c* direction [Fig. 2 ▸(*b*)]. There is one contact just below the sum of the van der Waals radii between two inter­locked helices: C3⋯C11^iii^ [symmetry code: (iii) *x*, −*y* + 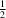, *z* − 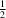] at 3.388 (2) Å [purple dotted lines on Fig. 2 ▸(*a*) and 3(*a*)]. The helix layers stack along *a* so that helices of the same handedness lie adjacent to each other, as expected for the space group *P*2_1_/*c* [Figs. 2 ▸(*b*); Miyata *et al.*, 2015 ▸]. The only close contact between the layers is a weak C9—H9⋯O2^ii^ [symmetry code: (ii), −*x* + 1, −*y* + 1, −*z* + 2] inter­molecular hy­dro­gen bond connecting helices of opposite handedness (Table 2 ▸). The geometrical parameters of this inter­action are consistent with a hy­dro­gen bond rather than packing effects.

**Figure 3:**
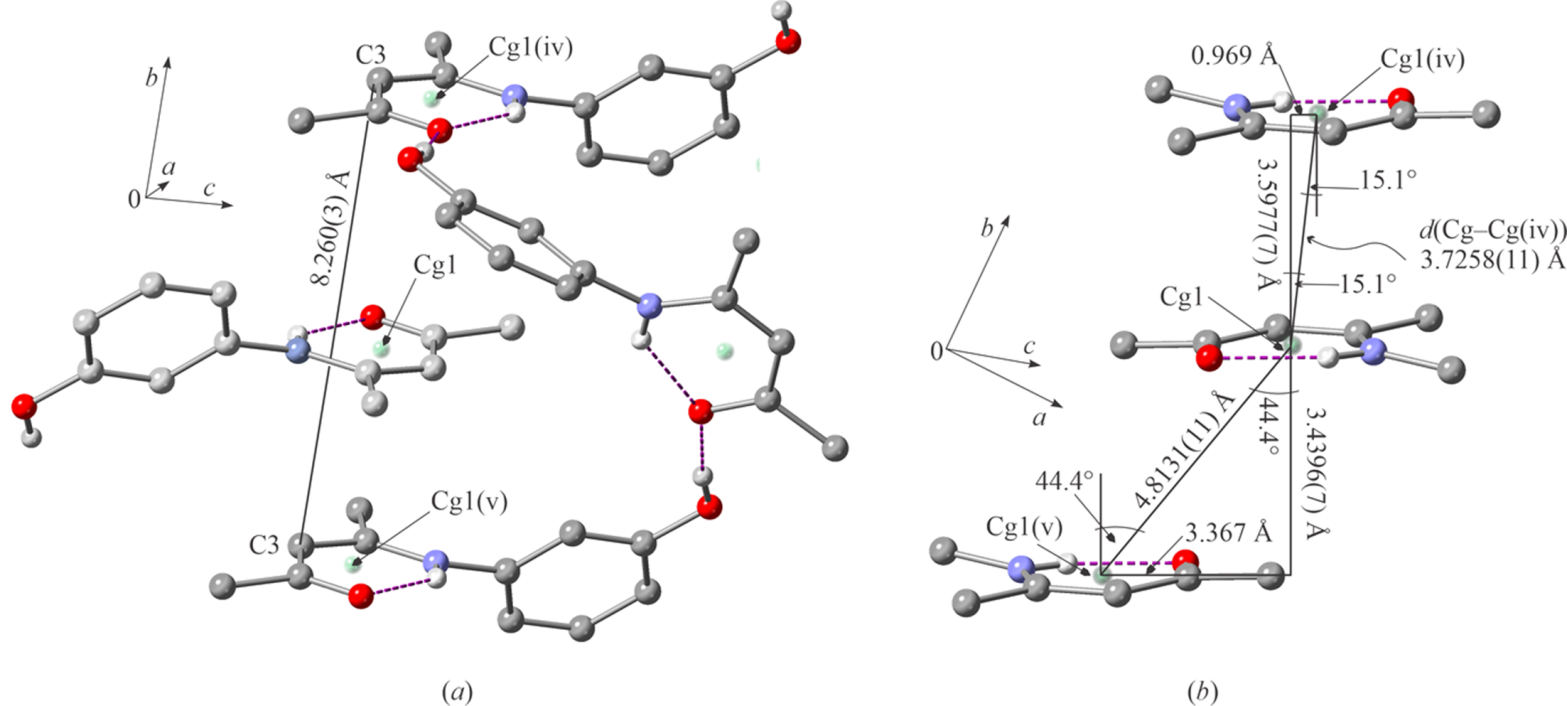
Helix groove and docking, showing (*a*) one groove and its docked mol­ecule, with the centroids of the HB rings indicated, and (*b*) centroid–centroid inter­action parameters.

The helix grooves, in which one mol­ecule of **I** ‘docks’, have an opening of 8.260 (3) Å and are com­posed of three mol­ecules [Fig. 3 ▸(*a*)]. The HB ring docking inter­actions were analysed using *PLATON* (Spek, 2020 ▸) and the findings are summarized in Fig. 3 ▸(*b*). The three rings, two from the groove and one from the docking mol­ecule, are stacked in an off-centre anti­parallel [α = 0.03 (8)°] fashion and are not equidistant. The *Cg*1⋯*Cg*1^iv^ [symmetry code: (iv) −*x*, −*y* + 1, −*z* + 1] pair displays a short centroid–centroid distance and only minimal lateral displacement, consistent with π–π stacking (Molčanov *et al.*, 2019 ▸). Similar parameters have already been reported for the RAHB rings (Blagojević Filipović *et al.*, 2019 ▸).

In contrast, although the the second centroid pair, *Cg*1⋯*Cg*1^v^ [symmetry code: (v) −*x*, −*y*, −*z* + 1], has a short inter­planar separation [3.4396 (7) Å], its long centroid–centroid distance [4.8131 (11) Å] and substantial slippage (3.367 Å) are not indicative of π–π stacking. Instead, this geometry corresponds to a slipped non-stacking inter­action dominated by electrostatic dipole–dipole contributions arising from the inter­action between C=O groups. Since carbonyl C atoms are planar (no pyramidalization is observed) and the C2⋯O1^v^ distance of 3.557 (2) Å is significantly above the 3.22 Å cut-off (Newberry & Raines, 2017 ▸), n→π* inter­actions between C=O groups can be exculded.

There are no significant contacts between benzene rings, with the closest centroid–centroid distance being 5.0134 (11) Å for *Cg*2⋯*Cg*2^vi^ [symmetry code: (vi) −*x* + 1, *y* − 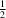, −*z* + 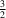].

## 4. Hirshfeld surface, fingerprint plots and lattice energy

**Figure 4:**
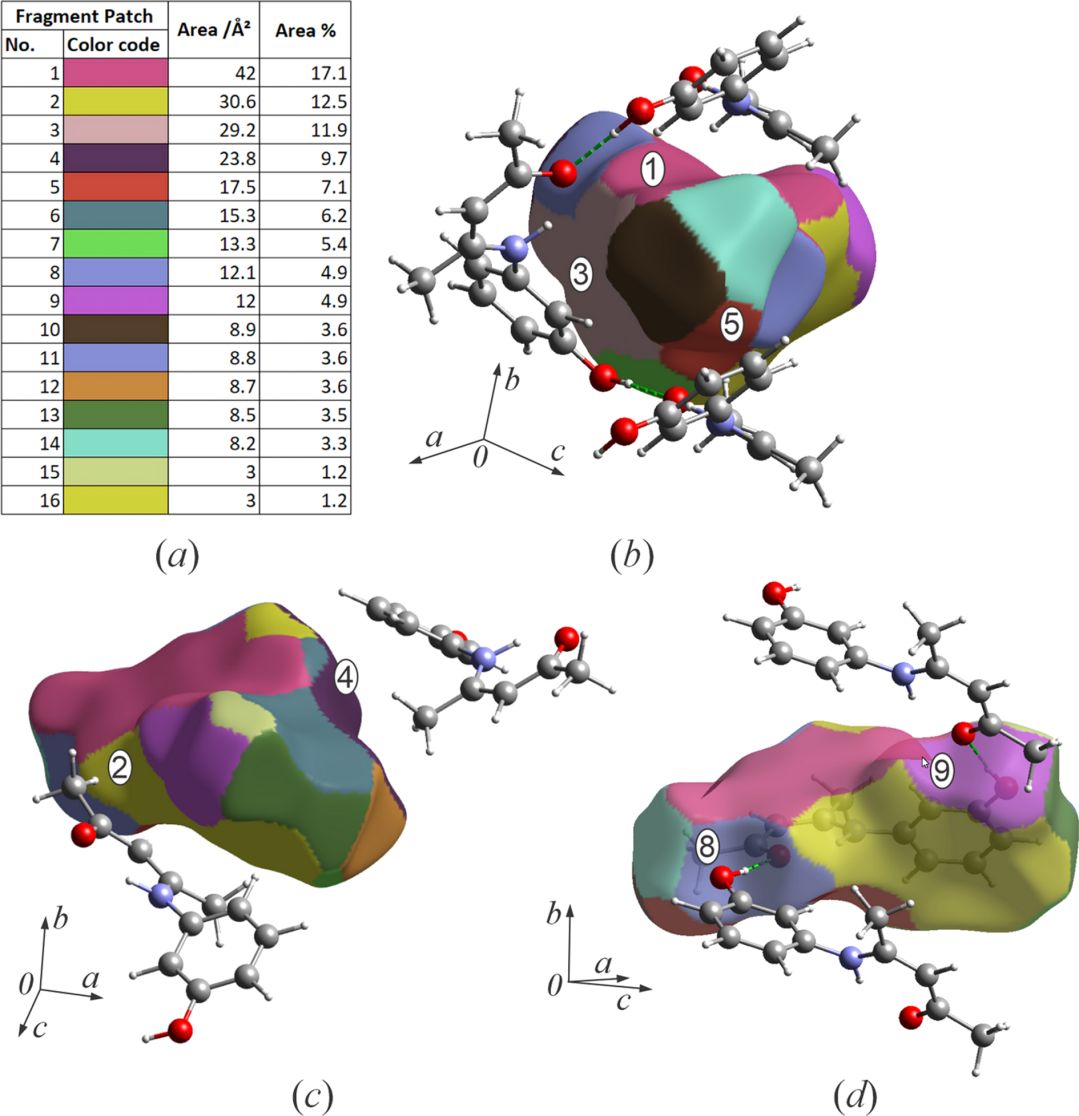
Hirshfeld surface with fragment patch analysis mapped for (*a*) 16 fragments and their contributions to the surface area, (*b*) helix groove contacts and their fragment patches, and (*c*) two large surface and (*d*) fragment patches corresponding to hy­dro­gen bonds within the helix.

To further analyse the inter­molecular inter­actions in the crystal structure of **I**, the Hirshfeld surface, with different functions mapped, and the fingerprint plots were generated using *CrystalExplorer 17.5* (Turner *et al.*, 2017 ▸). The fragment patch Hirshfeld surface reveals 16 nearest neighbours for each mol­ecule of **I** (Fig. 4 ▸). About 58% of the fragment patch surface belongs to five neighbours: three from the groove (patches 1, 3 and 5) and two from the mol­ecules of the next helix of the opposite handedness (patches 2 and 4), directed along the crystallographic *c* axis. The two hy­dro­gen-bonded mol­ecules of the same helix com­prise just under 10% of the surface combined (patches 8 and 9). The remaining nine contacts are distributed among layers along the crystallographic *a* axis.

**Figure 5:**
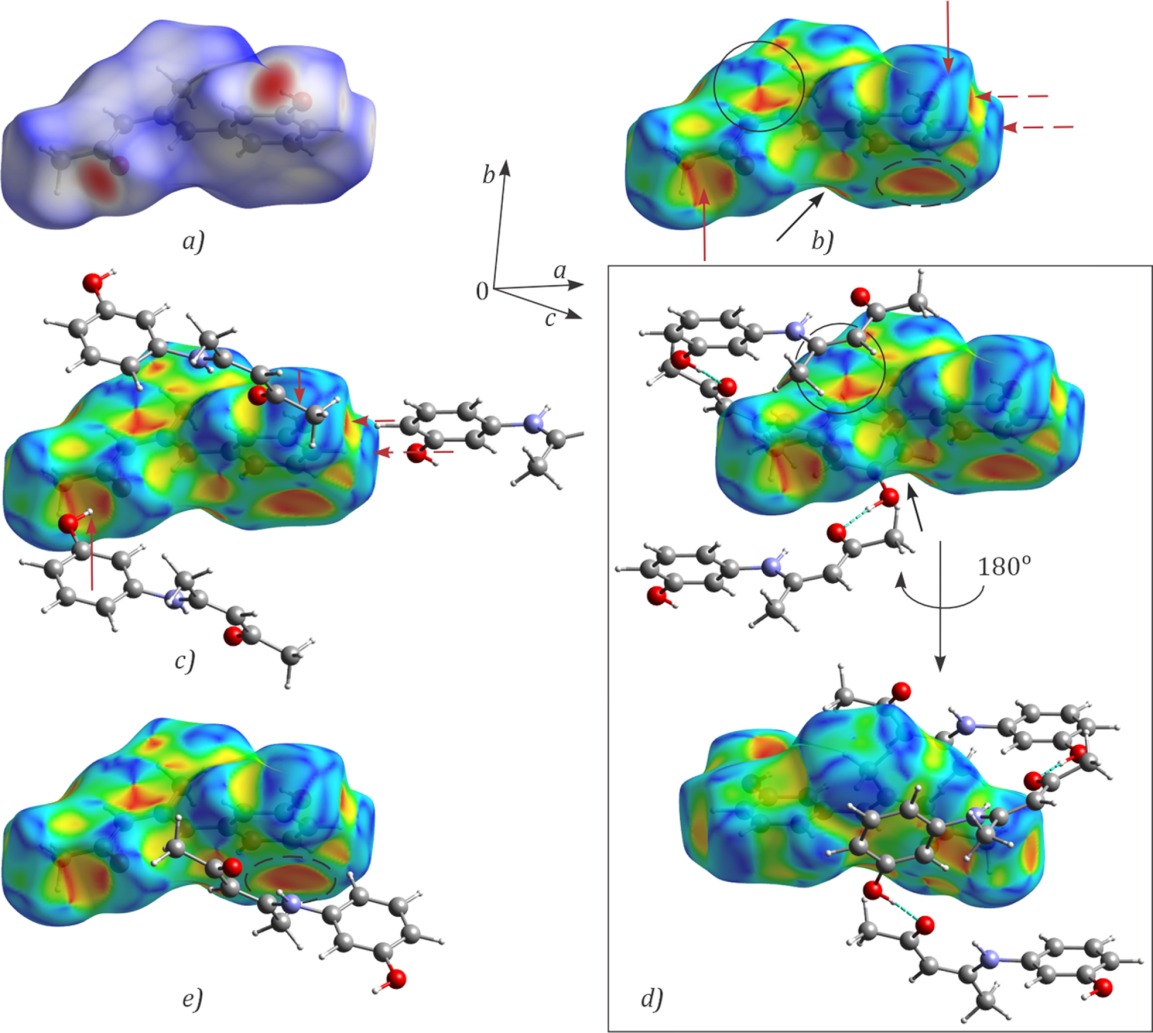
Hirshfeld surfaces, showing (*a*) *d*_norm_ surface, (*b*) shape index (*S*) surface, (*c*) hy­dro­gen bonding and *S* surface, (*d*) inter­actions within one helix groove showing π–π inter­actions between the HB rings and (*e*) C—H⋯π contacts.

The *d*_norm_ Hirshfeld surface [Fig. 5 ▸(*a*)], in the standard colour scheme, is dominated by white areas indicative of contacts close to the sum of the van der Waals radii. Two prominent red spots, at keto atom O1 and hydroxyl atom O2, highlight short contacts and coincide with the O—H⋯O hy­dro­gen bonds within a helix. Less prominent red areas correspond to the C9—H9⋯O2 contacts between helices of opposite handedness.

In contrast to the relatively featureless *d*_norm_, the shape index Hirshfeld surface, *S*, provides significant inter­action detail [Fig. 5 ▸(*b*)]. The *S* surface reveals ‘hollows’ (concave red regions) and ‘bumps’ (convex blue regions) on the surface where neighbouring mol­ecules inter­lock. A deep concave region at O1 and a high convex region at OH group [dark-red arrows; Fig. 5 ▸(*b*)] highlight the strong hy­dro­gen bonds responsible for helix formation [Fig. 5 ▸(*c*)]. A similar concave/convex pair is noticeable for the weaker C9—H9⋯O2 inter­helix inter­actions [dark dashed red arrows of Fig. 5 ▸(*b*)]. The bow-tie pattern of green and red/blue triangles [black circle; Fig. 5 ▸(*b*)], characteristic of stacking of flat rings and particularly π–π stacking (McKinnon *et al.*, 2004 ▸), supports the off-centre π–π stacking for the *Cg*1⋯*Cg*1^iv^ centroid pair [Fig. 5 ▸(*d*)]. The black arrows indicate convex regions where the second HB ring, defining *Cg*1^v^, docks. No bow-tie pattern is observed for these two HB rings. A deep concave region (dashed circle) below the benzene ring arises from inter­action with the C5 methyl group from a helix of opposite handedness [Fig. 5 ▸(*e*)]. The geometric parameters indicate a weak dispersion C—H⋯π inter­action involving part of the benzene ring, specifically the C8—C9 segment, with a C5—H⋯C9 distance of 2.871 Å (Nishio, 2011 ▸).

**Figure 6:**
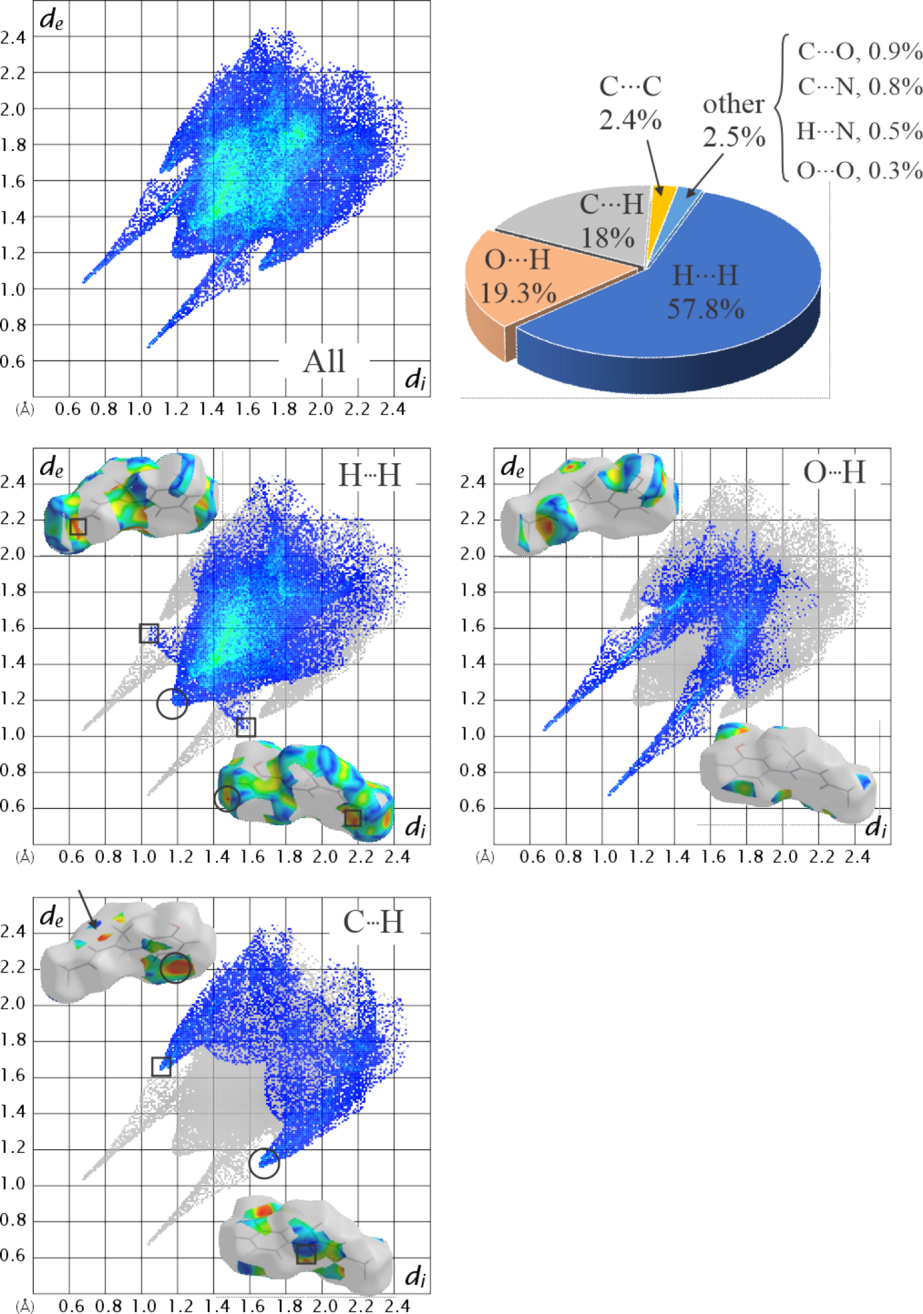
Distribution of contacts and fingerprint plots for **I**.

The fingerprint plot (Spackman & McKinnon, 2002 ▸) (Fig. 6 ▸) spans a broad range of *d*_i_ and *d*_e_ values, from about 1.2 to over 2.4 Å. The colour distribution, with a clear lack of red areas indicative of strong dominant inter­actions, suggests a significant proportion of small but non-zero contacts. The most prominent feature is a two-pronged shape of the plot, typical for hy­dro­gen-bonded systems (McKinnon *et al.*, 2004 ▸). Three inter­actions make up more than 95% of the total: H⋯H, O⋯H and C⋯H (pie chart in Fig. 6 ▸). The filtered fingerprint plots and *S* surfaces are shown in Fig. 6 ▸. The H⋯H contacts are absent around the HB and benzene rings. The small winged regions (marked with squares) correspond to contacts between mol­ecules of the same helix. A small tip at about 1.2 Å (circle) corresponds to the short contacts between helix layers. The central teal region of the filtered plot is dominated by contacts between stacked helices.

The O⋯H contacts span a broad range of *d*_i_/*d*_e_ values and the filtered *S* surface places them predominantly within a single helix. The tips of the two prongs correspond to the closest O—H⋯O contacts. The C⋯H fingerprint plot shows small wings (ellipses), characteristic of C—H⋯π inter­actions (McKinnon *et al.*, 2004 ▸). The tips of these wings correspond to the centre of a deep depression on the *S* surface, seen as a red region near the benzene ring. *PLATON* (Spek, 2020 ▸) ring analysis did not reveal any inter­action involving the delocalised π-system of the entire benzene ring. A detailed evaluation places the minimum of the concave (red) region around C9 and the corresponding convex (blue) region at H5*C* with points of closest approach of C9⋯H5*C*^vi^ [symmetry code: (vii) *x*, −*y* + 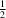, *z* + 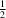] = 2.87 Å, C8⋯H5*C*^vi^ = 3.15 Å and C10⋯H5*C*^vii^ = 3.08 Å between the helices of the opposite handedness within one helix layer. The remaining important C⋯H contacts form a part of a bow-tie pattern with *d*_i_/*d*_e_ values in the range 1.8–2.0 Å. Another part of this pattern appears in the C⋯C inter­actions and lies in the teal-coloured region of the fingerprint plot. The tip of the plot corresponds to the short C3⋯C11^iii^ contact and appears as a small green patch on the corresponding Hirshfeld surface. Although they represent less than 2% of all contacts, O⋯C and N⋯C contacts com­plete the bow-tie pattern associated with HB ring stacking and are therefore essential for understanding the ring inter­actions.

**Figure 7:**
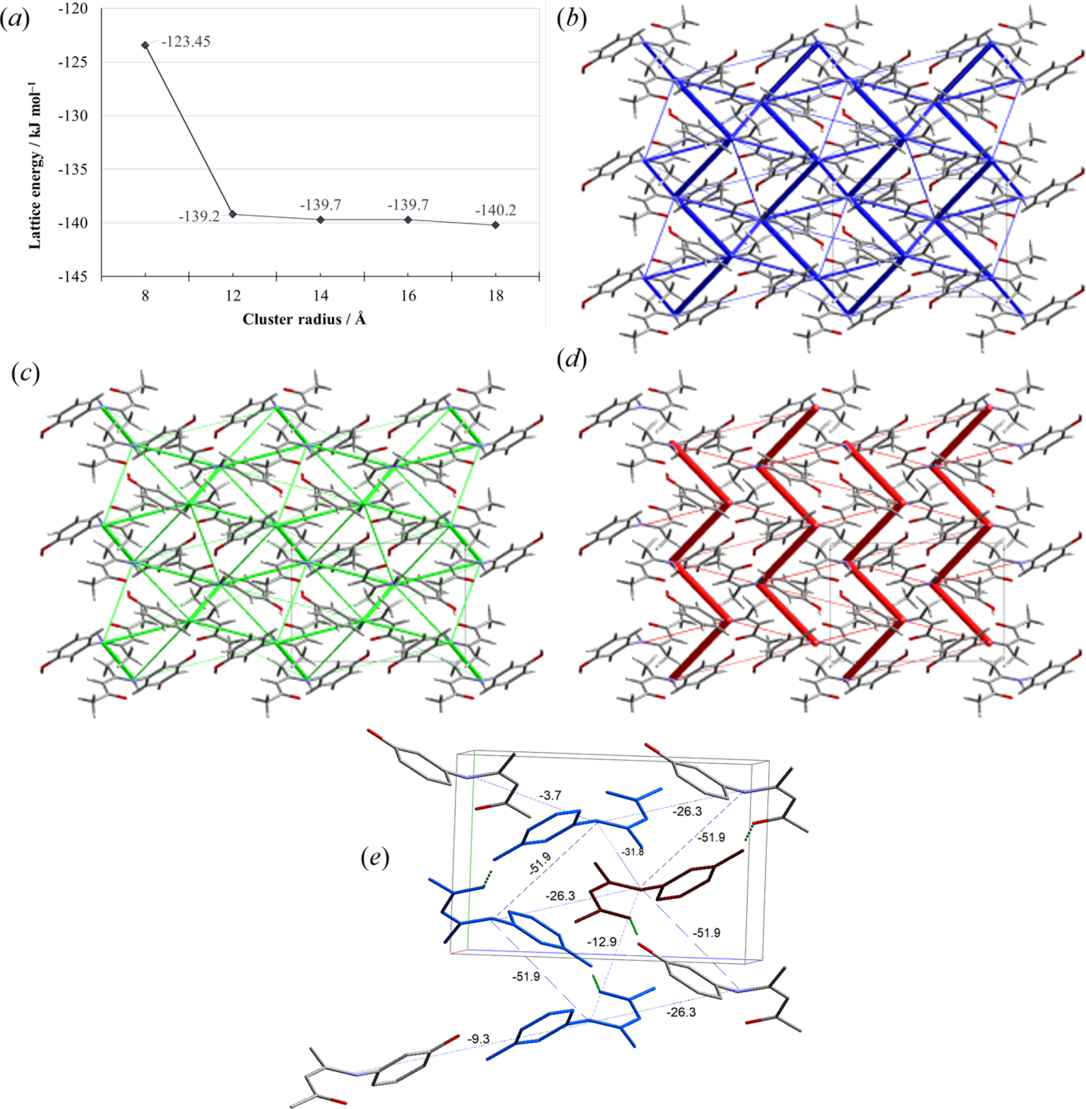
(*a*) Lattice energy convergence plot and (*b*) total energy, (*c*) dispersion, (*d*) Coulombic and (*e*) annotated total energy framework diagrams calculated using the CE-B3LYP/6-31G(p,d) benchmark electron-density model. The tube size is set at 40, and the energy cut-off at 3.00 kJ mol^−1^ for all framework diagrams. All diagrams are viewed along the crystallographic *a* axis.

The CE-B3LYP/6-31G(d,p) lattice energy, *E*_latt_ (Turner *et al.*, 2014 ▸), converged at −140 kJ mol^−1^ [Fig. 7 ▸(*a*)]. The principal contributor to *E*_latt_ is the dispersion term at −107.1 kJ mol^−1^, followed by the electrostatic and polarization terms at −90.16 and −18.35 kJ mol^−1^, respectively. The repulsion–exchange term destabilizes the lattice by +75.9 kJ mol^−1^.

The energy framework diagrams (Mackenzie *et al.*, 2017 ▸) show that the bulk of the Coulombic forces occur within a single helix [Fig. 7 ▸(*d*)], while the dispersion is almost equally distributed throughout the lattice but is somewhat stronger between inter­locking helices [Fig. 7 ▸(*c*)], consistent with previous findings on π-stacking (Molčanov *et al.*, 2019 ▸). As the annotated energies indicate, the strongest inter­acting is the pair of two hy­dro­gen-bonded molecules within a helix followed by the inter­actions within the helix grove [Fig. 7 ▸(*e*)].

## 5. Database survey

A search of the Cambridge Structural Database (CSD, Version 5.44, April 2023 update; Groom *et al.*, 2016 ▸) targeting structures derived from the condensation of acetyl­acetone and an aromatic amine provided 91 results (organic structures only). The closest relative to com­pound **I** is 4-[(2-hy­droxy­phen­yl)amino]­pent-3-en-2-one, the keto–imine derived from the condensation of acetyl­acetone and 2-amino­phenol. There are six deposits for this com­pound (all ortho­rhom­bic, space group *P*2_1_2_1_2_1_): CSD refcodes MEHTEY (Kabak *et al.*, 1998 ▸), MEHTEY1 (Chen *et al.*, 1999 ▸), MEHTEY2 (Rajnikant *et al.*, 2006 ▸), MEHTEY3 (Basu *et al.*, 2010 ▸), MEHTEY4 (Fatiha *et al.*, 2012 ▸) and MEHTEY5 (Salehi *et al.*, 2012 ▸). Inter­molecular hy­dro­gen bonding (also producing helical supra­molecular structure) has been discussed briefly in all cases. Salehi *et al.* (2012 ▸) also include a brief discussion on resonance-assisted hy­dro­gen bonds.

## 6. Synthesis and crystallization

Acetyl­acetone (2.3 mmol, 2.3 ml) was added dropwise to a stirred solution of 3-amino­phenol (2.3 mmol, 0.25 g) in 95% ethanol (10 ml). The reaction was stirred at ambient temperature for 45 min, covered and then left to stand. After two days, colourless crystals were collected giving an 88% yield (1.8 g) of the title com­pound.

### 6.1. Analytical data

^1^H NMR (CHCl_3_, δ from TMS): 2.08 (*s*, 3H, –CH_3_), 2.14 (*s*, 3H, –CH_3_), 5.20 (*s*, 1H, CH), ^13^C NMR (CHCl_3_, δ from TMS): 20.16, 28.46 (–CH_3_), 97.5 (CH, acac backbone), 111.5, 114.2, 115.7, 130.6, 138.8, 158.67 (benzene ring carbons), 163.0 (CN) and 169.1 (CO). Elemental analysis calculated (%) for C_11_H_13_NO_2_: C 69.09, N 7.32; found: C 69.07, N 7.56. MS–ESI: 190.097 (base peak, *M* – H^+^), 148.038 [*M* – C(=O)CH_3_]^.−^.

## 7. Refinement

Crystal data, data collection and structure refinement details are summarized in Table 3 ▸. The positional parameters of the H atoms bonded to N1 and O2, *i.e.* H1*N* and H1*O*, were refined while their displacement parameters were constrained in the usual manner. See the figures in the supporting information for the residual electron-density maps before and after the refinement of atoms H1*N* and H1*O*.

## Supplementary Material

Crystal structure: contains datablock(s) I, global. DOI: 10.1107/S2056989026004068/ex2099sup1.cif

Structure factors: contains datablock(s) I. DOI: 10.1107/S2056989026004068/ex2099Isup2.hkl

Supporting information file. DOI: 10.1107/S2056989026004068/ex2099Isup4.mol

Difference maps used to locate hdrogen atoms. DOI: 10.1107/S2056989026004068/ex2099sup3.pdf

Supporting information file. DOI: 10.1107/S2056989026004068/ex2099Isup5.cml

CCDC reference: 1818233

Additional supporting information:  crystallographic information; 3D view; checkCIF report

## Figures and Tables

**Table 1:** Selected geometric parameters (Å, °)

O1—C2	1.2627 (18)	C1—C2	1.503 (2)
N1—C4	1.3352 (19)	C2—C3	1.401 (2)
N1—C6	1.4253 (18)	C3—C4	1.379 (2)
N1—H1N	0.902 (18)	C4—C5	1.495 (2)
			
C4—N1—H1N	115.6 (11)	C4—C3—C2	125.24 (14)
C6—N1—H1N	116.6 (11)	N1—C4—C3	120.05 (14)
O1—C2—C3	123.26 (13)		

**Table 2:** Hydrogen-bond geometry (Å, °)

*D*—H⋯*A*	*D*—H	H⋯*A*	*D*⋯*A*	*D*—H⋯*A*
N1—H1N⋯O1	0.903 (18)	1.936 (18)	2.6651 (18)	136.6 (14)
O2—H1O⋯O1^i^	0.90 (2)	1.80 (2)	2.6886 (17)	173 (2)
C9—H9⋯O1^ii^	0.93	2.65 (2)	3.576 (2)	175 (2)

Symmetry codes: (i) 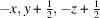; (ii) 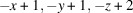.

**Table 3:** Experimental details

Crystal data	
Chemical formula	C_11_H_13_NO_2_
*M*_r_	191.22
Crystal system, space group	Monoclinic, *P*2_1_/*c*
Temperature (K)	295
*a*, *b*, *c* (Å)	10.109 (1), 8.2598 (8), 12.2074 (13)
β (°)	92.539 (4)
*V* (Å^3^)	1018.30 (18)
*Z*	4
Radiation type	Mo *K*α
μ (mm^−1^)	0.09
Crystal size (mm)	0.39 × 0.32 × 0.09

Data collection	
Diffractometer	Bruker SMART X2S
Absorption correction	Multi-scan (*SADABS*; Bruker, 2004 ▸)
No. of measured, independent and observed [*I* > 2σ(*I*)] reflections	12414, 2304, 1661
*R*_int_	0.037
(sin θ/λ)_max_ (Å^−1^)	0.649

Refinement	
*R*[*F*^2^ > 2σ(*F*^2^)], *wR*(*F*^2^), *S*	0.043, 0.131, 1.05
No. of reflections	2304
No. of parameters	135
H-atom treatment	H atoms treated by a mixture of independent and constrained refinement
Δρ_max_, Δρ_min_ (e Å^−3^)	0.16, −0.17

Computer programs: *XPREP* (Bruker, 2004 ▸), *SAINT* (Bruker, 2004 ▸) and *SHELXL2019* (Lübben *et al.*, 2019 ▸).

## supplementary crystallographic information

### (3Z)-4-[(3-Hydroxyphenyl)amino]pent-3-en-2-one Crystal data

**Table:**

C_11_H_13_NO_2_	*F*(000) = 408
*M**_r_* = 191.22	*D*_x_ = 1.247 Mg m^−^^3^
Monoclinic, *P*2_1_/*c*	Mo *K*α radiation, λ = 0.71073 Å
*a* = 10.109 (1) Å	Cell parameters from 3910 reflections
*b* = 8.2598 (8) Å	θ = 3.0–25.5°
*c* = 12.2074 (13) Å	µ = 0.09 mm^−^^1^
β = 92.539 (4)°	*T* = 295 K
*V* = 1018.30 (18) Å^3^	Prism, white
*Z* = 4	0.39 × 0.32 × 0.09 mm

### (3Z)-4-[(3-Hydroxyphenyl)amino]pent-3-en-2-one Data collection

**Table:**

Bruker SMART X2S diffractometer	1661 reflections with *I* > 2σ(*I*)
θ/2θ scans	*R*_int_ = 0.037
Absorption correction: multi-scan (SADABS; Bruker, 2004)	θ_max_ = 27.5°, θ_min_ = 2.0°
	*h* = −13→12
12414 measured reflections	*k* = −10→8
2304 independent reflections	*l* = −15→15

### (3Z)-4-[(3-Hydroxyphenyl)amino]pent-3-en-2-one Refinement

**Table:**

Refinement on *F*^2^	0 restraints
Least-squares matrix: full	Hydrogen site location: mixed
*R*[*F*^2^ > 2σ(*F*^2^)] = 0.043	H atoms treated by a mixture of independent and constrained refinement
*wR*(*F*^2^) = 0.131	*w* = 1/[σ^2^(*F*_o_^2^) + (0.0587*P*)^2^ + 0.1926*P*] where *P* = (*F*_o_^2^ + 2*F*_c_^2^)/3
*S* = 1.05	(Δ/σ)_max_ = 0.001
2304 reflections	Δρ_max_ = 0.16 e Å^−^^3^
135 parameters	Δρ_min_ = −0.17 e Å^−^^3^

### (3Z)-4-[(3-Hydroxyphenyl)amino]pent-3-en-2-one Special details

**Table:**

Geometry. All esds (except the esd in the dihedral angle between two l.s. planes) are estimated using the full covariance matrix. The cell esds are taken into account individually in the estimation of esds in distances, angles and torsion angles; correlations between esds in cell parameters are only used when they are defined by crystal symmetry. An approximate (isotropic) treatment of cell esds is used for estimating esds involving l.s. planes.

### (3Z)-4-[(3-Hydroxyphenyl)amino]pent-3-en-2-one Fractional atomic coordinates and isotropic or equivalent isotropic displacement parameters (Å^2^)

**Table:**

	*x*	*y*	*z*	*U*_iso_*/*U*_eq_	
O1	0.43035 (10)	0.81490 (15)	0.54180 (8)	0.0599 (3)	
O2	0.81010 (12)	0.44803 (18)	0.93832 (10)	0.0748 (4)	
H1O	0.728 (2)	0.407 (3)	0.9392 (16)	0.090*	
N1	0.65951 (13)	0.66563 (17)	0.58920 (10)	0.0518 (3)	
H1N	0.5874 (18)	0.719 (2)	0.6112 (13)	0.062*	
C1	0.35690 (18)	0.8435 (2)	0.35503 (14)	0.0701 (5)	
H1A	0.390076	0.818741	0.284480	0.105*	
H2A	0.348530	0.958690	0.362603	0.105*	
H3A	0.271802	0.793723	0.361572	0.105*	
C2	0.45131 (14)	0.77987 (19)	0.44339 (12)	0.0507 (4)	
C3	0.55872 (15)	0.68582 (19)	0.41245 (12)	0.0512 (4)	
H3	0.561171	0.656140	0.339084	0.061*	
C4	0.66112 (14)	0.63373 (18)	0.48205 (12)	0.0486 (4)	
C5	0.77347 (17)	0.5391 (2)	0.43852 (15)	0.0674 (5)	
H5A	0.743665	0.482666	0.373259	0.101*	
H5B	0.804881	0.462392	0.492717	0.101*	
H5C	0.843976	0.611567	0.421641	0.101*	
C6	0.76327 (14)	0.63947 (19)	0.67015 (12)	0.0477 (4)	
C7	0.88744 (15)	0.7068 (2)	0.65909 (15)	0.0627 (5)	
H7	0.906071	0.766290	0.596935	0.075*	
C8	0.98306 (15)	0.6842 (2)	0.74194 (15)	0.0642 (5)	
H8	1.067104	0.727533	0.734505	0.077*	
C9	0.95668 (14)	0.5991 (2)	0.83511 (14)	0.0566 (4)	
H9	1.021965	0.586059	0.890515	0.068*	
C10	0.83197 (14)	0.53259 (19)	0.84595 (13)	0.0494 (4)	
C11	0.73545 (13)	0.55284 (18)	0.76281 (12)	0.0461 (4)	
H11	0.651851	0.507906	0.769582	0.055*	

### (3Z)-4-[(3-Hydroxyphenyl)amino]pent-3-en-2-one Atomic displacement parameters (Å^2^)

**Table:**

	*U*^11^	*U*^22^	*U*^33^	*U*^12^	*U*^13^	*U*^23^
O1	0.0502 (6)	0.0799 (8)	0.0490 (6)	0.0070 (5)	−0.0035 (5)	−0.0044 (5)
O2	0.0536 (7)	0.1028 (11)	0.0667 (8)	−0.0156 (7)	−0.0118 (6)	0.0267 (7)
N1	0.0422 (6)	0.0632 (8)	0.0497 (7)	0.0027 (6)	−0.0027 (5)	−0.0012 (6)
C1	0.0693 (11)	0.0823 (13)	0.0570 (10)	0.0084 (9)	−0.0150 (8)	−0.0004 (9)
C2	0.0476 (8)	0.0552 (9)	0.0487 (8)	−0.0089 (7)	−0.0037 (6)	0.0023 (7)
C3	0.0526 (8)	0.0576 (10)	0.0433 (8)	−0.0071 (7)	0.0008 (6)	−0.0004 (7)
C4	0.0482 (8)	0.0473 (8)	0.0506 (8)	−0.0085 (6)	0.0035 (6)	0.0008 (7)
C5	0.0629 (10)	0.0683 (11)	0.0715 (11)	0.0053 (9)	0.0076 (8)	−0.0079 (9)
C6	0.0413 (7)	0.0477 (8)	0.0535 (8)	0.0018 (6)	−0.0041 (6)	−0.0034 (7)
C7	0.0510 (9)	0.0659 (11)	0.0706 (11)	−0.0122 (8)	−0.0031 (7)	0.0138 (8)
C8	0.0415 (8)	0.0670 (11)	0.0832 (12)	−0.0123 (7)	−0.0069 (8)	0.0117 (9)
C9	0.0407 (7)	0.0571 (10)	0.0707 (10)	0.0001 (7)	−0.0119 (7)	0.0022 (8)
C10	0.0430 (7)	0.0498 (9)	0.0550 (8)	0.0017 (6)	−0.0028 (6)	0.0013 (7)
C11	0.0350 (6)	0.0490 (8)	0.0541 (8)	−0.0020 (6)	0.0007 (6)	−0.0054 (7)

### (3Z)-4-[(3-Hydroxyphenyl)amino]pent-3-en-2-one Geometric parameters (Å, º)

**Table:**

O1—C2	1.2627 (18)	C5—H5A	0.9600
O2—C10	1.3528 (19)	C5—H5B	0.9600
O2—H1O	0.89 (2)	C5—H5C	0.9600
N1—C4	1.3352 (19)	C6—C11	1.378 (2)
N1—C6	1.4253 (18)	C6—C7	1.385 (2)
N1—H1N	0.902 (18)	C7—C8	1.380 (2)
C1—C2	1.503 (2)	C7—H7	0.9300
C1—H1A	0.9600	C8—C9	1.374 (2)
C1—H2A	0.9600	C8—H8	0.9300
C1—H3A	0.9600	C9—C10	1.387 (2)
C2—C3	1.401 (2)	C9—H9	0.9300
C3—C4	1.379 (2)	C10—C11	1.3865 (19)
C3—H3	0.9300	C11—H11	0.9300
C4—C5	1.495 (2)		
			
C10—O2—H1O	113.0 (13)	C4—C5—H5C	109.5
C4—N1—C6	127.50 (14)	H5A—C5—H5C	109.5
C4—N1—H1N	115.6 (11)	H5B—C5—H5C	109.5
C6—N1—H1N	116.6 (11)	C11—C6—C7	120.49 (13)
C2—C1—H1A	109.5	C11—C6—N1	118.50 (13)
C2—C1—H2A	109.5	C7—C6—N1	120.92 (14)
H1A—C1—H2A	109.5	C8—C7—C6	118.84 (16)
C2—C1—H3A	109.5	C8—C7—H7	120.6
H1A—C1—H3A	109.5	C6—C7—H7	120.6
H2A—C1—H3A	109.5	C9—C8—C7	121.38 (15)
O1—C2—C3	123.26 (13)	C9—C8—H8	119.3
O1—C2—C1	118.34 (15)	C7—C8—H8	119.3
C3—C2—C1	118.40 (14)	C8—C9—C10	119.50 (14)
C4—C3—C2	125.24 (14)	C8—C9—H9	120.3
C4—C3—H3	117.4	C10—C9—H9	120.3
C2—C3—H3	117.4	O2—C10—C11	122.46 (13)
N1—C4—C3	120.05 (14)	O2—C10—C9	117.82 (13)
N1—C4—C5	119.58 (14)	C11—C10—C9	119.72 (14)
C3—C4—C5	120.36 (14)	C6—C11—C10	120.06 (13)
C4—C5—H5A	109.5	C6—C11—H11	120.0
C4—C5—H5B	109.5	C10—C11—H11	120.0
H5A—C5—H5B	109.5		
			
O1—C2—C3—C4	−6.9 (3)	N1—C6—C7—C8	177.12 (16)
C1—C2—C3—C4	172.60 (15)	C6—C7—C8—C9	−1.1 (3)
C6—N1—C4—C3	−171.48 (14)	C7—C8—C9—C10	0.8 (3)
C6—N1—C4—C5	9.5 (2)	C8—C9—C10—O2	179.29 (15)
C2—C3—C4—N1	3.4 (2)	C8—C9—C10—C11	0.0 (3)
C2—C3—C4—C5	−177.56 (15)	C7—C6—C11—C10	0.1 (2)
C4—N1—C6—C11	−127.36 (17)	N1—C6—C11—C10	−176.49 (14)
C4—N1—C6—C7	56.1 (2)	O2—C10—C11—C6	−179.66 (15)
C11—C6—C7—C8	0.7 (3)	C9—C10—C11—C6	−0.4 (2)

### (3Z)-4-[(3-Hydroxyphenyl)amino]pent-3-en-2-one Hydrogen-bond geometry (Å, º)

**Table:**

*D*—H···*A*	*D*—H	H···*A*	*D*···*A*	*D*—H···*A*
N1—H1*N*···O1	0.903 (18)	1.936 (18)	2.6651 (18)	136.6 (14)
O2—H1*O*···O1^i^	0.90 (2)	1.80 (2)	2.6886 (17)	173 (2)
C9—H9···O1^ii^	0.93	2.65 (2)	3.576 (2)	175 (2)

Symmetry codes: (i) −*x*, *y*+1/2, −*z*+1/2; (ii) −*x*+1, −*y*+1, −*z*+2.
