# Supplementary material for: The crystal structure of (3Z)-4-[(3-hy­droxy­phen­yl)amino]­pent-3-en-2-one: helices and π-stacking inter­actions of hy­dro­gen-bridged rings
Source: Acta Crystallogr E Crystallogr Commun. 2026 May 7;82(Pt 6):588–95. doi: 10.1107/S2056989026004068 (PMC13239021; doi:10.1107/S2056989026004068)
Supplement: Supplementary file 4 [file e-82-00588-sup3.pdf]

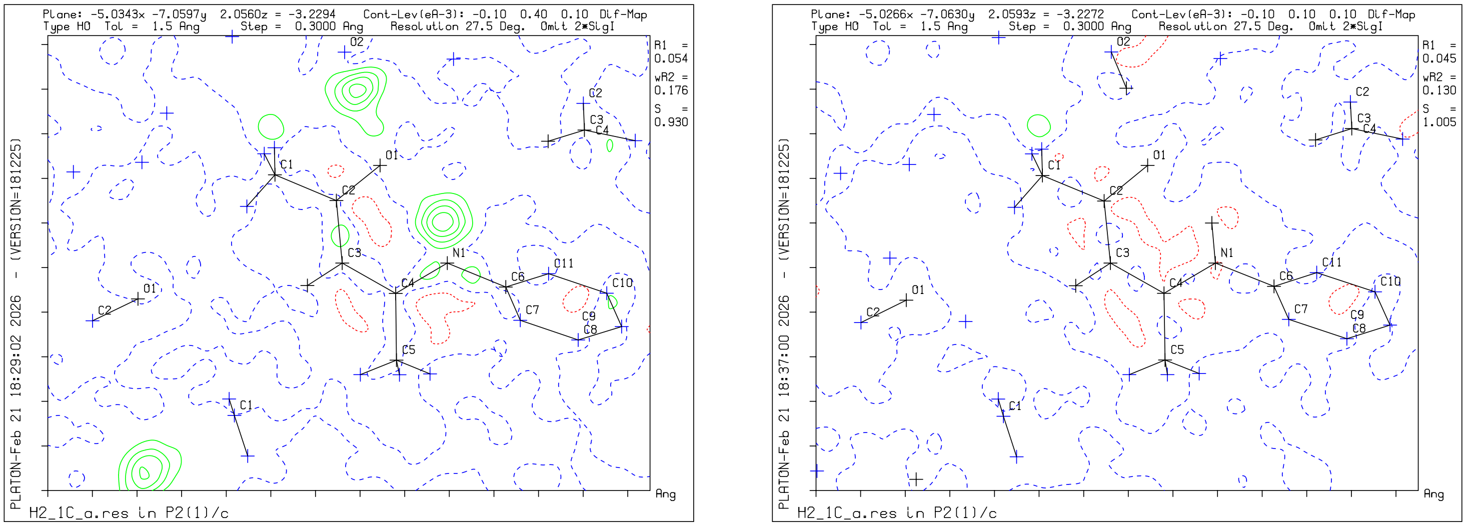

Difference maps in N1-C3-O1 plane: before (left) and after (right) H1N1 was introduced

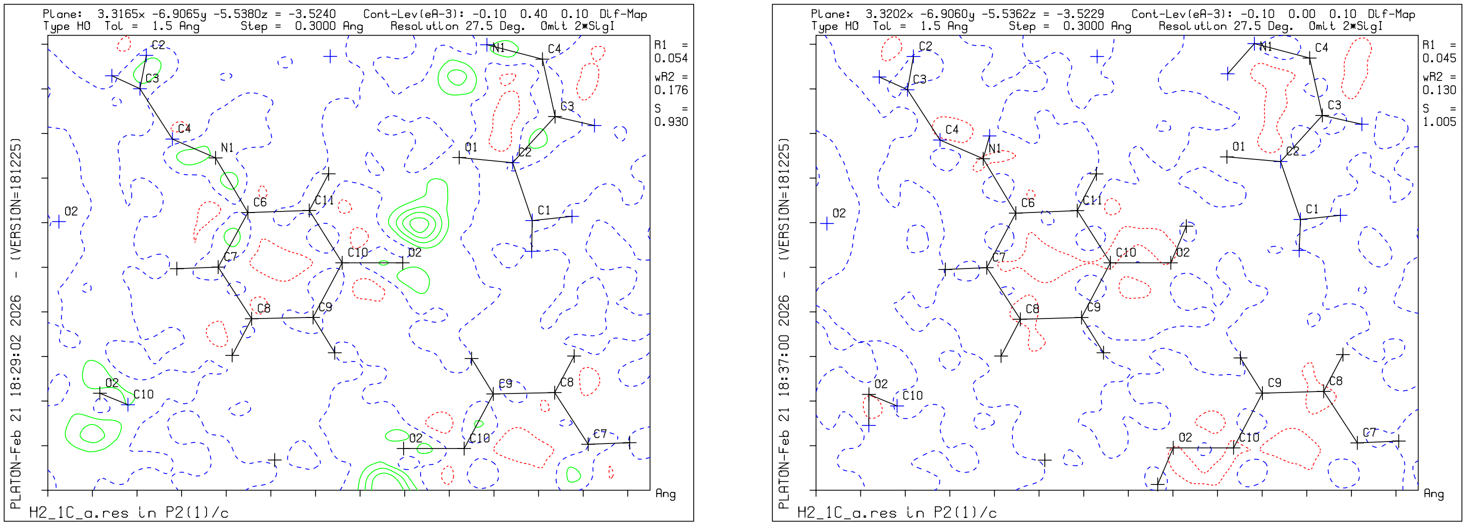

Difference maps in O2-C10-C11 plane: before (left) and after (right) H2O2 was introduced
